# Machine and Deep Learning Prediction Of Prostate Cancer Aggressiveness Using Multiparametric MRI

**DOI:** 10.3389/fonc.2021.802964

**Published:** 2022-01-13

**Authors:** Elena Bertelli, Laura Mercatelli, Chiara Marzi, Eva Pachetti, Michela Baccini, Andrea Barucci, Sara Colantonio, Luca Gherardini, Lorenzo Lattavo, Maria Antonietta Pascali, Simone Agostini, Vittorio Miele

**Affiliations:** ^1^ Department of Radiology, Careggi University Hospital, Florence, Italy; ^2^ “Nello Carrara” Institute of Applied Physics (IFAC), National Research Council of Italy (CNR), Sesto Fiorentino, Italy; ^3^ “Alessandro Faedo” Institute of Information Science and Technologies (ISTI), National Research Council of Italy (CNR), Pisa, Italy; ^4^ Department of Information Engineering (DII), University of Pisa, Pisa, Italy; ^5^ “Giuseppe Parenti” Department of Statistics, Computer Science, Applications(DiSIA), University of Florence, Florence, Italy; ^6^ Florence Center for Data Science, University of Florence, Florence, Italy

**Keywords:** prostate cancer, mpMRI prostate cancer aggressiveness, deep learning, machine learning, radiomics

## Abstract

Prostate cancer (PCa) is the most frequent male malignancy and the assessment of PCa aggressiveness, for which a biopsy is required, is fundamental for patient management. Currently, multiparametric (mp) MRI is strongly recommended before biopsy. Quantitative assessment of mpMRI might provide the radiologist with an objective and noninvasive tool for supporting the decision-making in clinical practice and decreasing intra- and inter-reader variability. In this view, high dimensional radiomics features and Machine Learning (ML) techniques, along with Deep Learning (DL) methods working on raw images directly, could assist the radiologist in the clinical workflow. The aim of this study was to develop and validate ML/DL frameworks on mpMRI data to characterize PCas according to their aggressiveness. We optimized several ML/DL frameworks on T2w, ADC and T2w+ADC data, using a patient-based nested validation scheme. The dataset was composed of 112 patients (132 peripheral lesions with Prostate Imaging Reporting and Data System (PI-RADS) score ≥ 3) acquired following both PI-RADS 2.0 and 2.1 guidelines. Firstly, ML/DL frameworks trained and validated on PI-RADS 2.0 data were tested on both PI-RADS 2.0 and 2.1 data. Then, we trained, validated and tested ML/DL frameworks on a multi PI-RADS dataset. We reported the performances in terms of Area Under the Receiver Operating curve (AUROC), specificity and sensitivity. The ML/DL frameworks trained on T2w data achieved the overall best performance. Notably, ML and DL frameworks trained and validated on PI-RADS 2.0 data obtained median AUROC values equal to 0.750 and 0.875, respectively, on unseen PI-RADS 2.0 test set. Similarly, ML/DL frameworks trained and validated on multi PI-RADS T2w data showed median AUROC values equal to 0.795 and 0.750, respectively, on unseen multi PI-RADS test set. Conversely, all the ML/DL frameworks trained and validated on PI-RADS 2.0 data, achieved AUROC values no better than the chance level when tested on PI-RADS 2.1 data. Both ML/DL techniques applied on mpMRI seem to be a valid aid in predicting PCa aggressiveness. In particular, ML/DL frameworks fed with T2w images data (objective, fast and non-invasive) show good performances and might support decision-making in patient diagnostic and therapeutic management, reducing intra- and inter-reader variability.

## 1 Introduction

Prostate cancer (PCa) is the most frequent male malignancy and the third cause of cancer death in European men with significant consequences for healthcare systems (1). In biopsy-naïve men the clinical suspicion of PCa is based on an elevated serum prostate-specific antigen (PSA) level and/or an abnormal digital rectal examination. However, multiparametric (mp) MRI is strongly recommended before biopsy (2, 3), because the latter procedure, if it’s not targeted, has low sensitivity and specificity, thus leading to underdiagnosis of clinically significant PCa and to overdiagnosis of non clinically significant PCa.

Indeed, over the last decades, mpMRI has become increasingly valuable for the detection and staging of PCa, gaining a key role in the diagnostic pathway (4) and apparent advantages compared to the systematic transrectal ultrasonography-guidedbiopsy (TRUSGB) (5). Firstly, it can rule out non clinically significant (cs) PCa, thus reducing the number of unnecessary prostate biopsies and overdiagnosis. Secondly, it also enables targeted biopsies of suspected lesions, allowing better risk stratification (6, 7). However, performing many mpMRI acquisitions and reporting is an essential challenge for the uroradiological community. Efforts have been made in creating and constantly updating the Prostate Imaging Reporting and Data System (PI-RADS) guidelines that recommend a systematized mpMRI acquisition and define a global standardization of reporting (8). In particular, the PI-RADS score assigns a numerical value in the interval [1 - 5] to the suspected lesion, correlated with the probability of the lesion being a cs malignancy. However, there is still a lack of consensus on the detailed aspects of mpMRI acquisition protocols and the radiologists’ requirements for reading the examinations (e.g., experience prerequisites for independent reporting are still absent) (9). For these reasons, the assessment of csPCa is still based on visual, qualitative evaluation with individual level reports, and the diagnostic process is relatively slow, subjective, and dependent on the experience level of the radiologist. For example, fewer cases with PI-RADS score equal to 3, which corresponds to an indeterminate probability of csPCa, have been reported from expert readers compared to non-expert ones (6, 10). Additionally, the PI-RADS score measures the probability of malignancy and not the PCa aggressiveness. Thus, the biopsy is still needed to assess the csPCa aggressiveness by measuring the International Society of Urological Pathology (ISUP) Grade Group (GG) and the Gleason Score (GS) (11). The assessment of PCa aggressiveness is fundamental for patient management because lower-grade cancers grow more slowly and are less likely to spread toward other organs than high-grade cancers (4, 12–18). Therefore, assessing the tumor aggressiveness is an essential step in guiding the urologist’s therapeutic choice, together with the TNM stage and other factors, e.g., individual life expectancy, general state and health and preference of the individual patient. Quantitative assessment of lesion aggressiveness on mpMRI might reinforce MRI importance, role, and value in PCa diagnostic, prognostic and monitoring pathway, providing the radiologist with an objective and noninvasive tool and thus decreasing intra- and inter-reader variability (19). This would permit the urologist to accordingly choose and/or modify the management approach, optimizing quality of life of many patients. In biopsy naïve patients, those with non clinically significant PCa may directly avoid or postpone any treatment or may begin active surveillance, thus reducing the number of biopsies and lessening the risk of overdiagnosis and overtreatment. During active surveillance, in a protocol-mandated future perspective, together with PSA and clinical data, quantitative mpMRI and relative analyses could actively bring out lesion progression, maybe reducing the need of re-biopsies.

In this view, radiomics deals with the extraction of high-dimensional quantitative features from clinical images using advanced mathematical algorithms (20, 21). These imaging features can be related to physiological and clinical outcomes to identify possible associations (22). Due to their high dimensionality, Machine Learning (ML) methods are increasingly being incorporated into radiomic studies (22). At the same time, Deep Learning (DL) algorithms can learn valuable features from raw images directly showing promising results in various computer vision tasks and are emerging as a disruptive alternative to feature engineering-based techniques (23). In recent years, many studies used radiomics in combination with ML/DL models on mpMRI data of PCa patients with the ultimate goal of assisting the radiologist in the diagnostic workflow (19). The frontrunners focused primarily on the proof of concept of radiomics and ML/DL techniques to detect prostate lesions or differentiate malign from benign lesions (24–35). More recent literature investigated the clinically relevant problem of identifying high-grade vs. low-grade tumors (19). Despite the promising results, previous literature presents critical issues that prevent a direct comparison among the different results and a reliable application in daily clinical practice. Specifically, from a clinical point of view, the outcomes have been obtained by predicting at the MR slice level (28, 36–38) rather than at the lesion level, as it is good practice in clinical reporting. These results, therefore, can not be deployed in a real clinical context. Moreover, only a few studies have used an independent cohort to evaluate the obtained models on external data (27, 39–45). Methodologically, previous works usually lack sufficient details to make them reproducible and seem to suffer from data leakage, reporting overly optimistic results (28, 36–38, 44–51). In mpMRI of PCa, the most common data leakage causes are i) inappropriate validation schemes, where the data split is based on the single MRI slice and not on the whole lesion and/or patient (37, 38), ii) the absence of a nested process for the hyperparameters optimization (28, 36, 37, 44–51). Moreover, in previous works the authors trained a small number of specific algorithms, the selection of which is not been adequately motivated (36, 38, 44–46, 49–51).

For these reasons, in this paper, we have investigated the potential role of several ML and DL frameworks in predicting PCa aggressiveness from mpMRI data, using a computational workflow that prevents the previously mentioned issues. Indeed, we trained, validated, and tested ML/DL frameworks (i.e., the concatenation of preprocessing steps and the actual classification models) using a patient-based nested validation scheme, to perform, at the same time, hyperparameters optimization, models selection, and the estimation of generalization performance on unseen data, without data leakage, at lesion level. Our cohorts contained overall 112 PCa patients, whose peripheral lesions obtained a PI-RADS score ≥ 3. All the ML and DL frameworks have been developed on data acquired following PI-RADS 2.0 guidelines. To evaluate the learning capabilities of ML/DL frameworks on data acquired with different protocols, a PI-RADS 2.1 cohort has been used as additional test set and the entire ML/DL analysis workflow has been repeated on a multi-PI-RADS dataset, constructed by merging images acquired following the PI-RADS 2.0 and 2.1 guidelines.

## 2 Material and Methods

### 2.1 Participants and MRI Examinations

**Figure 1:**
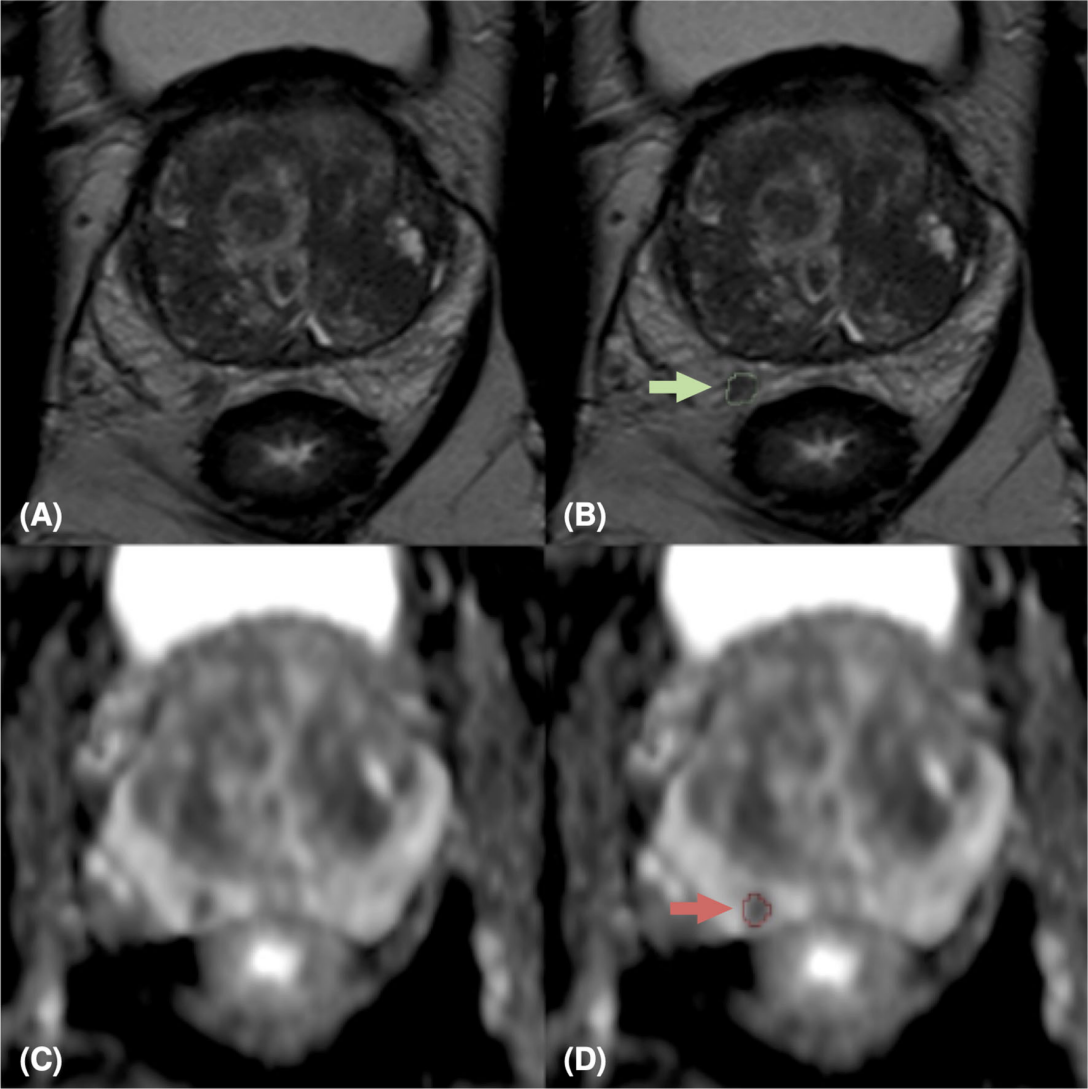
MpMRI of a 76-years old patient with indeterminate mpMRI result (PI-RADS=4), PSA=14 ng/ml, GS=4+4 (ISUP 4). MpMRI zoomed images containing the target lesion, respectively axial T2-weighted image **(A)**, ADC map **(C)**, and their relative lesion segmentations **(B, D)**. The green **(B)** and red **(D)** arrows point out the segmented lesion in T2 and ADC images, respectively.

**Figure 2:**
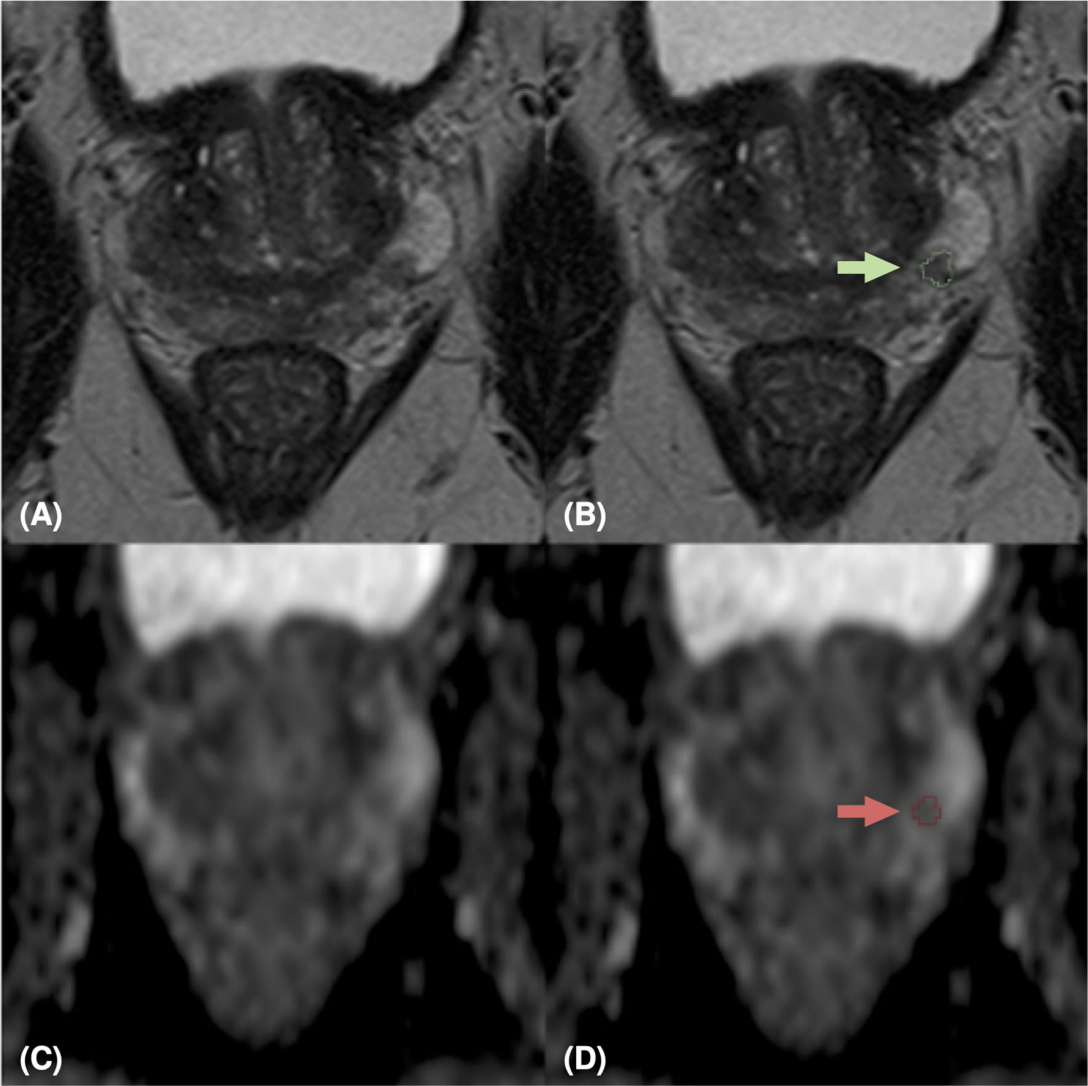
MpMRI of a 65-years old patient with indeterminate mpMRI result (PI-RADS=3), PSA=5.49 ng/ml, GS=3+4 (ISUP 2). MRI zoomed images containing the target lesion, respectively axial T2-weighted image **(A)**, ADC map **(C)**, and their relative lesion segmentations **(B, D)**. The green **(B)** and red **(D)** arrows point out the segmented lesion in T2 and ADC images, respectively.

**Figure 3:**
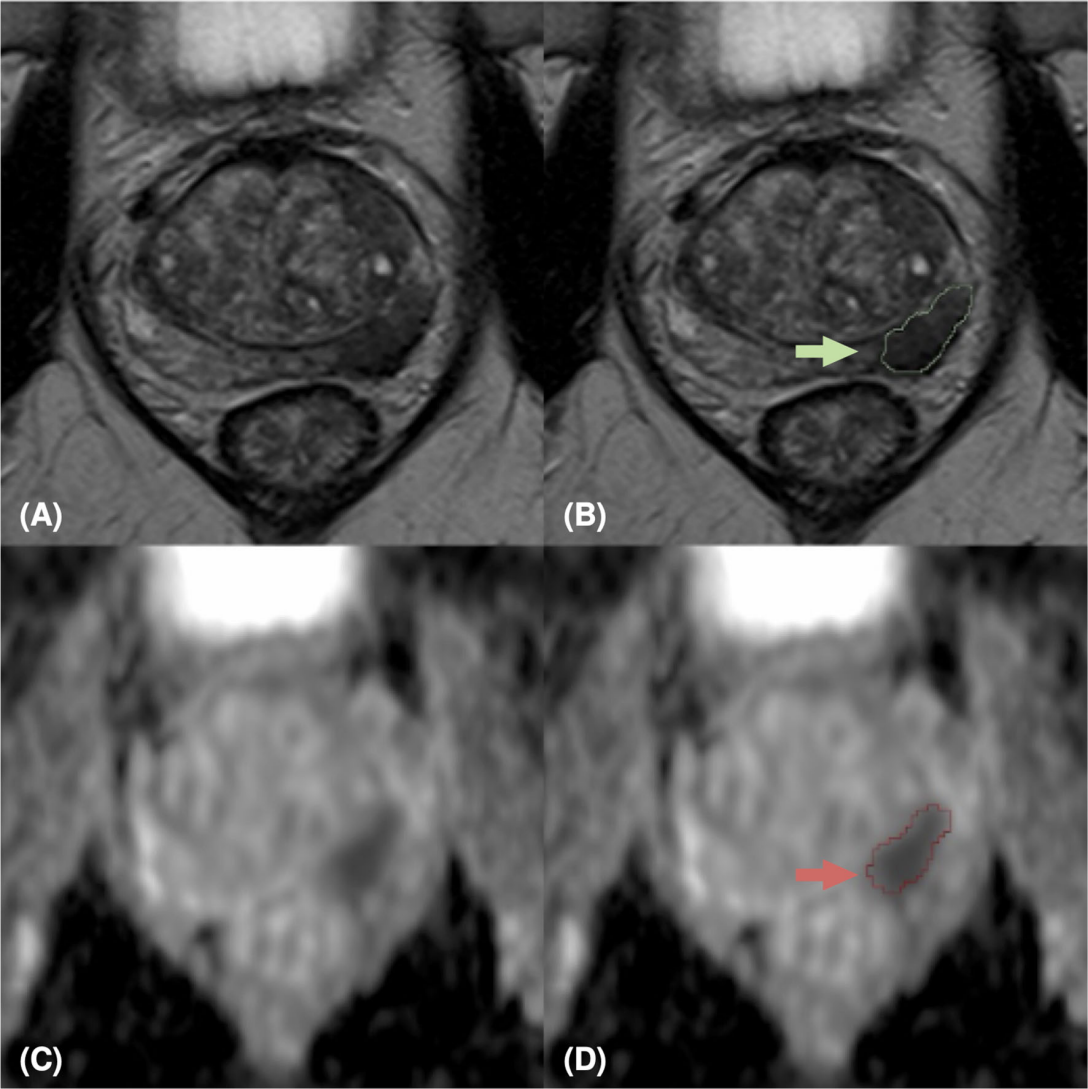
MpMRI of a 69-years old patient with positive mpMRI result (PI-RADS=5), PSA level=7 ng/ml, GS=4+4 (ISUP 4). MRI zoomed images containing the target lesion, respectively axial T2-weighted image **(A)**, ADC map **(C)**, and their relative lesion segmentations **(B, D)**. The green **(B)** and red **(D)** arrows point out the segmented lesion in T2 and ADC images, respectively.

Our study is monocentric and observational. Between June 2018 and December 2019, we enrolled 112 histopathologically confirmed peripheral zone PCa patients who underwent free-hand transperineal MRI/US fusion-guided targeted biopsy based on a positive/indeterminate mpMRI result, i.e., PI-RADS score ≥ 3. All mpMRI examinations were performed using a 1.5 T MR scanner equipped with an anterior pelvic phased-array 18-channel coil and a posterior spine phased-array 16-channel coil (Magnetom Aera, Siemens Medical Systems, Erlangen, Germany). Eighty-five patients have been acquired following the PI-RADS 2.0 guidelines, while the other 27 have been examined with mpMRI protocols according to the latest guidelines of PI-RADS 2.1. Aware that the PI-RADS 2.1 guidelines did not change the requirements for T2w acquisitions, in this study, the T2w image acquisition protocol was also changed, to obtain a better quality image while adhering to the guidelines (details in **Supplementary Section 1.1**). In our study, we focused on the most clinically relevant images, i.e., T2w images and ADC maps derived from multi-b Diffusion Weighted (DW) images. Three uro-radiologists (SA, EB, LM) with, respectively, 10, 6, and 2 years of experience in prostatic radiology, evaluated all MRI exams and assigned the PI-RADS scores in consensus. The lesions were manually segmented on T2w images and ADC maps. We show examples of mpMR images and segmentations in **Figures 1**–**3**. Histopathological examination, performed on the specimen taken during biopsy, provided the PCa aggressiveness by measuring the GS and the ISUP GG, which better reflects PCa biology (52). Because of different prognostic significance, we have identified low-grade (LG) lesions [i.e., with ISUP GG ≤ 2 and GS ≤ 7 (3 + 4)] and high-grade (HG) lesions [i.e., with ISUP GG≥3 and GS≥7 (4 + 3)]. Our final PI-RADS 2.0 cohort was composed of 85 patients and 103 lesions, while PI-RADS 2.1 cohort was formed by 27 patients and 29 lesions (details in **Table 1**).

**Table 1:** Descriptive statistics of our cohorts. GS ≤ 3+4 is equivalent to ISUP GG ≤ 2, and GS≥4+3 corresponds to ISUP GG≥3.

	PI-RADS 2.0 cohort	PI-RADS 2.1 cohort
# patients	85	28
Age (years) (mean (STD)	66.72 (7.58)	68.64 (5.71)
# lesions	103 (76 with GS ≤ 3+4,	29 (21 with GS ≤ 3+4,
	27 with GS≥4+3)	8 with GS≥4+3)
PI-RADS score (median ± IQR)	4 ± 0*	4 ± 0.625*
PSA (ng/ml) (mean (STD))	8.34 (8.20)*	5.43 (2.48)*

* Indicates significant differences (p-value < 0.5 at Mann-Whitney test) between PI-RADS 2.0 and PI-RADS 2.1 cohorts.

GS, Gleason score; IQR, interquartile range; ISUP GG, ISUP/WHO Grading Group; PI-RADS, Prostate Imaging – Reporting and Data System; PSA, Prostate-Specific Antigen, STD, standard deviation.

### 2.2 Prediction of PCa Aggressiveness Using Machine Learning Techniques

In this study, we predicted the lesion aggressiveness (i.e., LG vs. HG) from T2w images and ADC maps. In particular, we exploited two different strategies: (i) conventional ML techniques to identify the predictive power of the radiomic features extracted from each lesion; (ii) DL architectures to extract complex and aggressiveness-related features directly from raw images. All the frameworks presented below were trained, validated, and tested starting from either T2w images only, ADC maps only, or the combination of the two acquisition modalities, from now on referred as T2w/ADC/T2w+ADC. We have detailed the experimental tests of ML/DL analysis in **Supplementary Section 1.2**.

#### 2.2.1 ML Analysis: Radiomics Features Extraction and Models

For each slice, we computed a total of 95 2D radiomics features in compliance with the Image Biomarker Standardisation Initiative (IBSI) (details in **Supplementary Section 1.3** and **Tables S1**, **S2**). In the training set only, we performed a data augmentation by oversampling the minority class (i.e., the HG group) to reduce the effect of the imbalanced dataset (ratio LG : HG=2:1). Accordingly, we applied either Adaptive Synthetic (ADASYN) (53) or Synthetic Minority Oversampling TEchnique (SMOTE) (54) and its variants, i.e., the BorderlineSMOTE (55), SVMSMOTE (56) with default parameters.

Since, in general, it is not possible to define *a priori* the best class of ML models in a given problem (57), we used several popular and powerful supervised classes of ensemble classifiers. They are able to combine the predictions of several base classifiers with the aim of improving generalizability and robustness over a single ML classifier. In particular, we used three averaging methods, i.e., Bagging (58), Random Forests (59), and randomized decision trees (a.k.a. extra-trees) (60). Also, we employed three boosting methods, i.e., AdaBoost (61), Gradient Boosting (62), and eXtreme Gradient Boosting (XGBoost) (63). A grid consisting of different combinations of hyperparameters to optimize has been defined for each algorithm (**Table S3**). We detailed the training, validation and testing of the ML frameworks in *Section 2.2.3*.

#### 2.2.2 DL Analysis: Data Preprocessing and CNN Architectures

In this study, we designed Convolutional Neural Networks (CNN) working on 2D data. We cropped each slice containing the tumor tissue around the center of the lesion yielding T2w images of 64 x 64 pixels and ADC maps of 44 x 44 pixels. Hereinafter, we will name *C-DS* (Cropped-Dataset) this dataset of 2D images. In addition, from these cropped images, we generated the *L-DS* (Lesion-Dataset) obtained by exploiting the segmentation mask provided by radiologists (i.e., setting to zero the intensity of pixels not belonging to the tumor lesion). The former dataset was intended to provide a model robust against segmentation inaccuracies, and to assess whether the tissue around the lesion contributed with helpful information (e.g., exploiting the contrast between tumor and benign tissue as a potentially significant feature). The latter allowed a consistent comparison with radiomics-based analysis and ML approach. We adopted data augmentation techniques to compensate for class-imbalance. In the training set only, we added new instances of original HG images, generated by rotation (angle randomly sampled in the range [-25, 25] degrees), translation (horizontal and vertical shift randomly sampled in the range [-0.02, 0.02] image width/height, respectively), and vertically and horizontally flip.

Since it is not possible to define *a priori* the architecture of the CNN that best performs a specific task, a two-step optimization strategy has been completed: a grid search to select the most promising network architecture and a random search to optimize the hyperparameters (details in **Supplementary Section 1.4** and **Tables S4–S6**). The output of the grid and random searches was a set of six CNN architectures with their best hyperparameters: three of them trained with L-DS T2w/ADC/T2w+ADC images and the others trained with C-DS T2w/ADC/T2w+ADC images. Moreover, we added two Attention Gates (AGs) to the three optimal architectures trained on C-DS T2w/ADC/T2w+ADC images (64). AGs help the CNN to focus on target structures by suppressing irrelevant regions and highlighting important ones with the goal of improving prediction performance (64). Also, AGs showed to be more efficient when placed on layers handling higher-level and more specific features (64). Hence, we tested different placements for the AGs, considering only the middle and the final layers of the architecture (details in **Supplementary Section 1.4**).

#### 2.2.3 Training, Validation, and Test of ML/DL Frameworks

In this work we used the term *framework* to refer to concatenation of the different steps of our analysis. Indeed, ML approach involved data standardization, data augmentation, and classifier estimation. At the same time, DL consisted of the data augmentation followed by the network that performs the classification (**Figure S1**).

**Figure 4:**
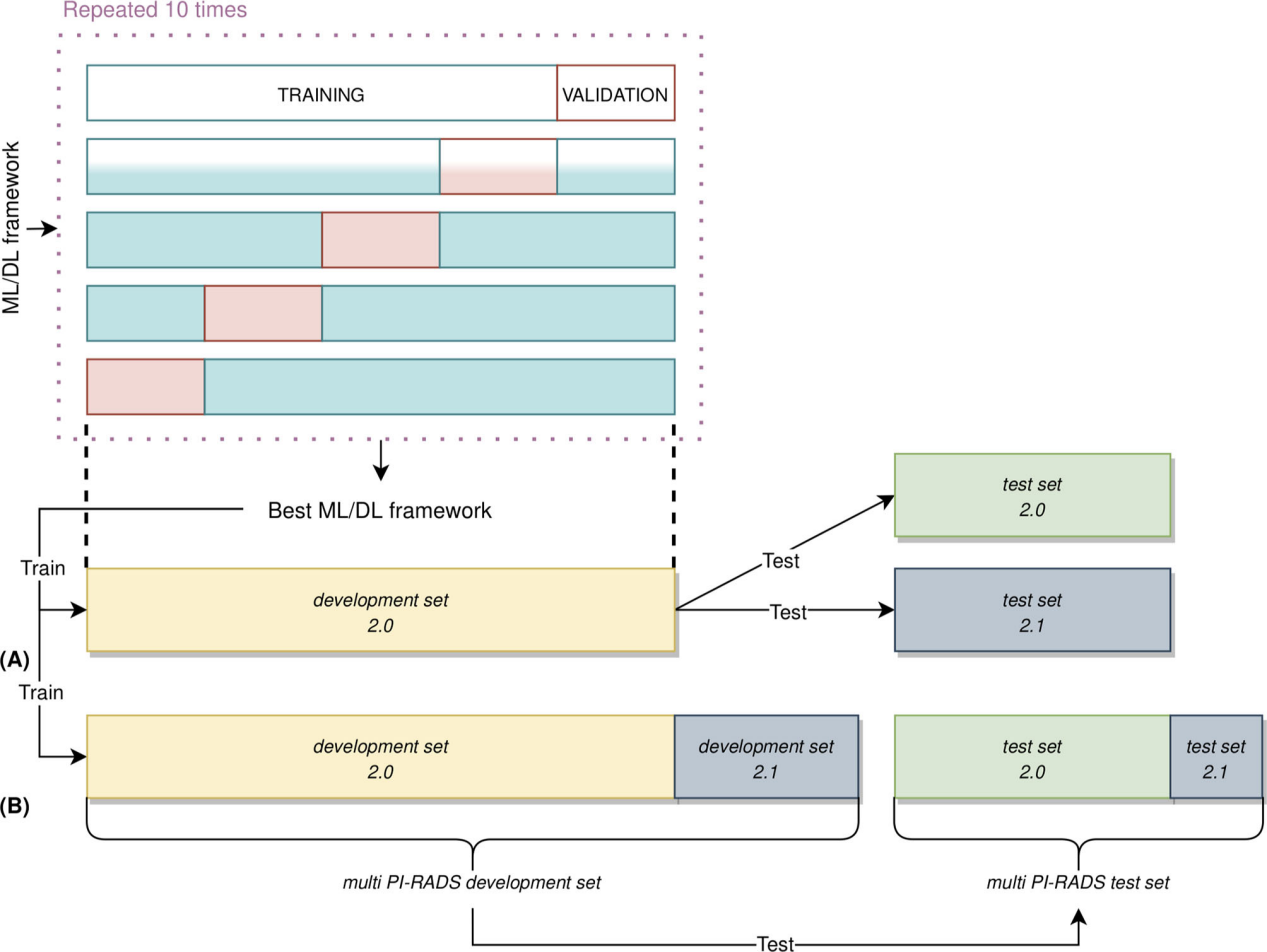
Nested validation scheme in our ML/DL analysis. **(A)** On development set 2.0 only, 5-fold CV was used to identify the best performing framework, along with performing hyperparameters optimization. The best performing ML/DL framework was then used to train the final framework on the entire development set 2.0. This framework was then evaluated on an unseen test set 2.0 and test set 2.1, independently. **(B)** The best performing framework was still trained on multi PI-RADS development set (i.e., the union of development set 2.0 and development set 2.1 and evaluated on unseen multi PI-RADS test set (i.e., the union of test set 2.0 and test set 2.1).

For each acquisition modality, the ML/DL frameworks have been trained, validated, and tested using the following approach (**Figure 4**): 87% of the entire PI-RADS 2.0 cohort was considered as the *development set 2.0*, and the remaining 13% as the independent *test set 2.0*. The PI-RADS 2.1 cohort has been used in two different ways: firstly, we have considered the entire cohort as an independent test set, and, secondly, we have split it in *development set 2.1*, containing images of 19 PCa patients, and *test set 2.1*, with images of eight patients. This last division allowed to create two new multi PI-RADS datasets. The *multi PI-RADS development set* consisted of the *development 2.0* merged with *development 2.1* and *multi PI-RADS test set*, composed of *test set 2.0* and *test set 2.1*.

On the development set 2.0, each model has been trained and validated using a patient-level, stratified 5-fold cross-validation (CV) strategy to perform framework selection along with hyperparameters optimization (65). In the 5-fold CV, 4 folds were used as training set while the other one as the validation set (details in **Supplementary Section 1.5**). The CV procedure has been repeated ten times using different random splits to deal with the variability in framework and hyperparameters selection derived from a specific data split (66). We have computed the average and standard deviation of the Area Under the Receiver Operating curve (AUROC) across all repetitions to get the final scores. The best frameworks were chosen based on the average AUROC scores in the validation set. Finally, the best frameworks were retrained on the whole development set 2.0 and tested on the unseen test set 2.0 and test set 2.1, independently. Moreover, the same ML/DL framework has been trained on the multi PI-RADS development set and evaluated on the multi PI-RADS test set. In order to consider the variability in the AUROC measurement due to the randomness of our test data, we drew additional bootstrap test sets of size equal to the original test set’s one (67, 68). Briefly, we randomly sampled, with replacement, the original test set data at the lesion level. The bootstrap sampling was repeated 1000 times, and the optimal ML/DL framework was then tested on each of these new additional bootstrap sample test sets, resulting in a series of AUROC values. We computed the median, 5^th^ and 25^th^ percentiles of AUROC values. Details about DL frameworks retraining has been reported in **Supplementary Section 1.5**.

## 3 Results

**Figure 5:**
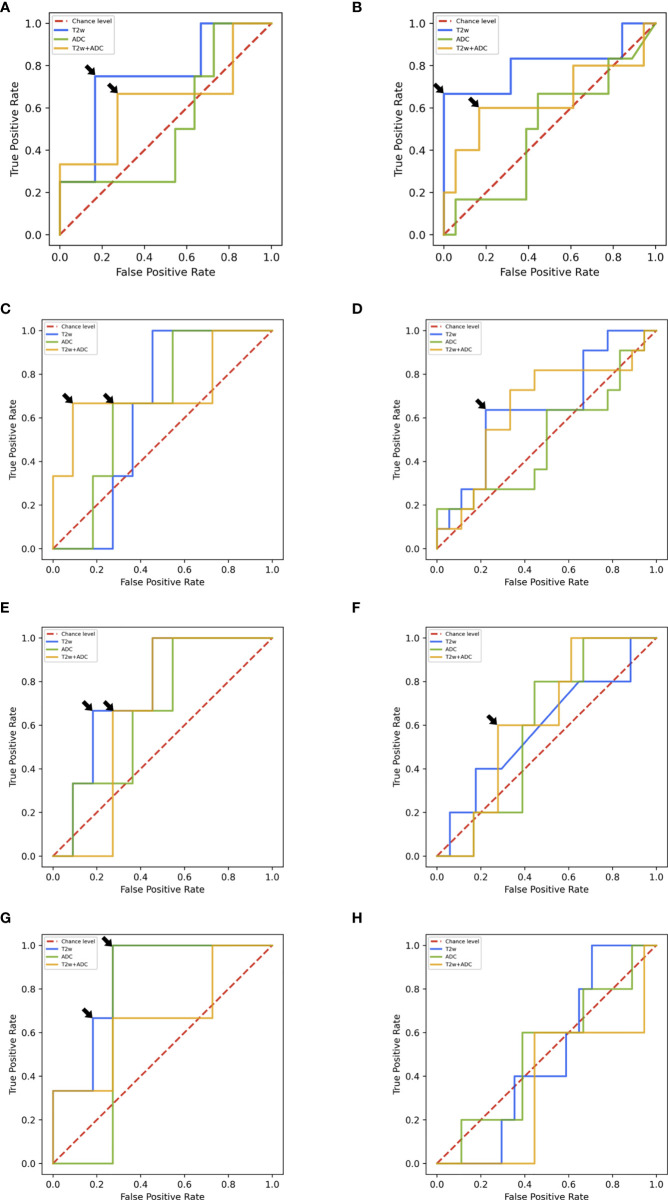
**(A)** ROC curves of ML frameworks on the test set 2.0. **(B)** ROC curves of ML frameworks on the multi PI-RADS test set. **(C)** ROC curves of DL AG-free CNN trained on L-DS test set 2.0. **(D)** ROC curves of DL AG-free CNN trained on L-DS multi PI-RADS test set. **(E)** ROC curves of DL AG-free CNN trained on C-DS test set 2.0. **(F)** ROC curves of DL AG-free CNN trained on C-DS multi PI-RADS test set. **(G)** ROC curves of DL AG CNN trained on C-DS test set 2.0. **(H)** ROC curves of DL AG CNN trained on C-DS multi PI-RADS test set.

In the following, we report the performance of the best ML/DL frameworks selected in the validation set in terms of median AUROC for T2w/ADC/T2w+ADC data. **Table 2** and **Figures 5A–H** summarizes all the prediction performances.

**Table 2:** AUROC values of ML and DL analyses for T2w/ADC/T2w+ADC.

Framework	Test set	T2w	ADC	T2w+ADC
ML	2.0	0.750 [0.500, 1]	0.531 [0.250, 0.75]	0.625 [0.167, 1]
	multi PI-RADS	0.795 [0.615; 1]	0.500 [0.300; 0.715]	0.682 [0.455; 1]
AG-free DL on L-DS	2.0	0.667 [0.385, 0.849]	0.667 [0.355, 0.905]	0.727 [0.231, 1]
	multi PI-RADS	0.750 [0.568, 0.945]	0.714 [0.445, 0.883]	0.752 [0.564, 0.872]
AG-free DL on C-DS	2.0	0.775 [0.478, 1]	0.667 [0.392, 0.903]	0.700 [0.455; 0.858]
	multi PI-RADS	0.524 [0.200, 0.818]	0.547 [0.393, 0.780]	0.574 [0.286, 0.819]
AG DL on C-DS	2.0	0.875 [0.639, 1]	0.750 [0.455, 0.911]	0.667 [0.301, 1]
	multi PI-RADS	0.500 [0.278, 0.717	0.463 [0.234, 0.817]	0.288 [0.09, 0.529]

The AUROC values are reported as median [5^th^ percentile, 95^th^ percentile]. AG: attention gate; C-DS, cropped dataset; DL, deep learning; L-DS, lesion dataset; ML, machine learning.

### 3.1 ML Analysis

On the test set 2.0, the framework trained with radiomic features extracted from T2w images showed the best AUROC value, i.e., 0.750. In particular, for specificity = 0.833, sensitivity was 0.750 (**Figure 5A**). On the other hand, the framework trained on ADC maps gave an AUROC no better than the chance level. The framework trained on T2w+ADC data obtained AUROC = 0.625). Notably, for specificity = 0.727, sensitivity was 0.667 (**Figure 5A**). However, this performance did not exceed that achieved by the framework trained on T2w data alone. All these frameworks, trained on radiomics features extracted from PI-RADS 2.0 T2w/ADC/T2w+ADC images, were tested also using radiomics features extracted from PI-RADS 2.1 T2w/ADC/T2w+ADC images. They all achieved AUROC values no better than the chance level (details in **Table S7** and **Figure S2A**). For the ML frameworks trained, validated and tested un the multi PI-RADS test set, the behaviour of the performance was similar to that observed on the test set 2.0. Indeed, the framework trained with radiomic features extracted from T2w images showed the best AUROC value, i.e., 0.795. In particular, for specificity = 1.000, sensitivity was 0.666 (**Figure 5B**). Conversely, the framework trained on ADC maps gave an AUROC no better than the chance level. The framework trained on T2w+ADC data showed good performances (i.e., AUROC = 0.682). Notably, for specificity = 0.883, sensitivity was 0.600 (**Figure 5B**). However, this performance was not better than that obtained by training the framework on T2w images only.

Eventually, in **Table S8**, we detailed the characteristics of the best performing ML frameworks, along with their optimal hyperparameters.

### 3.2 DL Analysis

On the test set 2.0, the AG framework trained with the C-DS T2w images achieved the best performance, i.e., AUROC = 0.875. In particular, for specificity = 0.727, sensitivity was 1.000 (**Figure 5G**). For ADC maps, the best framework was the AG CNN trained with the C-DS, and achieved AUROC = 0.727. Notably, for specificity = 0.727, sensitivity was 1.000 (**Figure 5G**). Conversely, for T2w+ADC images, the best framework was the AG-free CNN trained on the L-DS, and achieved AUROC = 0.750. In particular, for specificity = 0.909, sensitivity was 0.667 (**Figure 5C**). In line with ML results, the DL framework trained on T2w images achieved the overall best performance. Consistently with ML results, the frameworks trained on PI-RADS 2.0 T2w/ADC/T2w+ADC images and tested on PI-RADS 2.1 T2w/ADC/T2w+ADC images gave an AUROC around the chance level (details in **Table S7** and **Figures S2B–D**). The best performing DL framework trained, validated and tested on the multi PI-RADS test set was the AG-free CNN fed with L-DS T2w+ADC images, achieving AUROC = 0.752, but the AG-free CNN trained with L-DS T2w images only showed good performance equally (i.e., AUROC = 0.750). In particular, for specificity = 0.778, sensitivity was 0.600 (**Figure 5D**).

Finally, in **Table S9**, we reported the characteristics of the best performing DL architectures, along with their optimal hyperparameters. The optimal AG-free and AG CNN architectures trained with C-DS T2w images, and the AG-free CNN fed with C-DS T2w+ADC images have been represented in **Figures S3–S5** respectively.

## 4 Discussion

This study aims to predict PCa aggressiveness using ML/DL techniques on quantitative mpMRI data. In particular, we focused on peripheral lesions considered radiologically indeterminate or malignant (i.e., with PI-RADS ≥ 3), and examined according to PI-RADS 2.0 and 2.1 guidelines. Firstly, we extracted radiomic features from T2w images and ADC maps of lesions and fed them to various ML models. Then, we trained several DL architectures to directly analyze raw images. Both workflows were carried out following a rigorous validation scheme for hyperparameters optimization and estimation of the generalization capabilities on unseen data.

**Figure 6:**
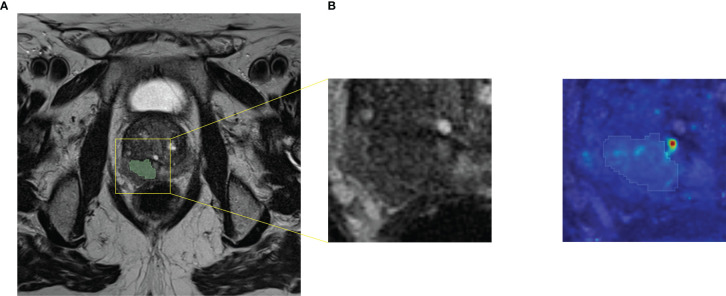
Axial T2w MR image of a PCa lesion with PI-RADS=5 and GS=4+5. **(A)** Original image together with its cropped version of size 64x64. We fed AG DL frameworks with the cropped images, i.e. the C-DS images. **(B)** Attention map obtained from the best performing AG DL framework fed with T2w C-DS images, superimposed to the original cropped version of the T2w image and its segmentation.

The performances achieved by both ML and DL frameworks trained on T2w data were higher than those obtained by training on ADC maps or T2w+ADC data. The best ML framework gave a median AUROC equal to 0.795. Notably, for specificity = 1.000, sensitivity was 0.666, while the best DL architecture showed an AUROC equal to 0.875. In particular, for specificity = 0.727, sensitivity was 1.000. The better performances on T2w images may be due to the higher spatial resolution and dynamic range of T2w images, compared to ADC maps. Conversely, information derived from ADC maps seems to be potentially confounding for ML/DL frameworks. Although extremely useful for visual assessment, the combination of the two acquisition modalities does not appear to improve the training of ML/DL frameworks. Intriguingly, the best performance of the ML framework was obtained on the multi PI-RADS test set. To the best of our knowledge, this is the first time that a ML framework has been trained and tested on mpMRI data acquired with different acquisition protocols without any data harmonization. Our results suggest that differences introduced in radiomic features due to different T2w image acquisition protocols do not hinder the ML models learning. In contrast, the performance of DL frameworks on the multi PI-RADS test set was worse (except for AG-free CNN trained with L-DS data), likely due to the fewer PI-RADS 2.1 images compared to those according to PI-RADS 2.0. Indeed, DL frameworks might need more PI-RADS 2.1 images during the training phase to improve performance on the multi PI-RADS test set. The best performance of the DL framework was obtained by an AG CNN architecture trained on T2w images containing both the lesion and the surrounding tissue. The inclusion of AGs layers seems, in most cases, to focus the attention of the entire architecture on the contour of the lesion, i.e., in the transition zone between the tumor and healthy tissue (**Figure 6**). The additional information provided by the out-of-lesion tissue might has improved the learning of the DL framework, since it is known that MRI consistently underestimates the size and extent of PCa lesions (69, 70). The ML/DL frameworks trained on PI-RADS 2.0 T2w/ADC/T2w+ADC data were tested on images acquired following PI-RADS 2.1 guidelines, but all the performances were around the chance level. Arguably, the features extracted are strictly related to the image acquisition parameters making these frameworks immature for a direct large-scale clinical use.

The results of our study are in line with previous works, which report ML/DL models’ AUROC values in the interval [0.70 - 0.93] (28, 38, 44, 46–51). Albeit feature selection is out of the scope of our work because it would be necessary to study also its stability to vary training data and model selection, we analyzed radiomic feature importance, providing insight into the data and the models. For the ML frameworks trained with radiomic features extracted from T2w images, those who got a better AUROC value, the highest predictors of PCa were textural features (see **Supplementary Figures S6** and **S7** for details). This result confirms that the analysis of quantitative features (not visible to the radiologist’s naked eye) by ML techniques effectively contributes to the prediction of PCa aggressiveness and could, in the future, be performed in a clinical context.

From a methodological point of view, we worked with 2D data (i.e., 2D radiomic features and single axial slices for T2w/ADC/T2w+ADC images) because some lesions were so small as to be visible on only one axial slice. Considering the lesion a 3D volume, regardless of the actual space occupied by the segmentation as other authors have done (39–42, 44, 71), seemed to be an overly forced assumption in most cases. In addition, though, we reported the test sets’ performance on a lesion level. This choice allowed us to obtain results that are in line with the radiologist assessment in a clinical context (19). Some authors worked with 2D data, but reported the final performance on slice level, making their results difficult to compare to ours (36, 72–75). It is well-known that ML/DL techniques perform better when trained on large and balanced datasets (76, 77). We addressed the imbalance problem of our datasets by using methods of randomly oversampling the minority class for tabular and imaging data (78). The most frequent algorithms used in the literature for PCa aggressiveness prediction were ML logistic regression and DL CNN, but without enough details about why those models were chosen (19). Previous literature showed insufficient awareness of the impact of the selected framework and hyperparameters on the generalizability of the results (79). Since it is not possible to define *a priori* which is the best performing ML/DL framework in a specific case (57), we opted for a data-driven approach. We reduced the potential overfitting caused by developing the ML/DL frameworks on a relatively small cohort by adopting a rigorous validation setup. We split T2/ADC/T2w+ADC data into two groups: the development and the test set. The development set was then further divided into training and validation set to perform framework selection along with hyperparameters optimization using a stratified 5-fold CV scheme. We repeated the 5-fold CV ten times to compensate for the sampling bias issue. It is essential to underline that, unlike other works (74, 75), we performed a patient-based splitting, and thus avoiding results inflated by the phenomenon of data leakage (80). We used the average value of the AUROC in the validation set to select the best ML/DL frameworks, and evaluated the generalizability on test sets allocated in the hold-out procedure. A strict comparison between ML and DL approaches was out of the scope of our work. It is true that DL techniques have more significant potential than the combination of hand-crafted features extraction and ML analysis for extensive datasets with thousands or even millions of instances. However, this is rarely the case of medical image analyses, where datasets are usually made up of hundreds/thousands of patients at best (81).

Our study has some limitations. Firstly, due to our rigorous approach to collect MRI data with PI-RADS score within the range [3 - 5] of peripheral zone PCa only, our sample size was relatively small, though similar to previous studies (72, 82, 83). Secondly, our study was monocentric. Given the complexity of assessing PCa aggressiveness from radiological images, monocentric acquisitions allowed us to keep the quantitative imaging as comparable as possible across patients. Experience showed that in PCa mpMRI, larger and multicenter/multi-scanner/multi-protocols datasets are difficult to find (84). Therefore, it is not surprising that the images used by most previous studies were generated using a single scanner or two scanners of the same vendor in one center.

In conclusion, the quantitative assessment of mpMRI might provide the radiologist with an objective and noninvasive tool for supporting the imaging work-up of patients affected by PCa. Actually, both ML and DL techniques applied on mpMRI seem to be a valid aid in predicting PCa aggressiveness. In particular, ML/DL frameworks fed with T2w images data (objective, fast and non-invasive) show good performances and might support decision-making in patient diagnostic and therapeutic management, decreasing intra- and inter-reader variability.

## Data Availability Statement

The raw data supporting the conclusions of this article will be made available by the authors, without undue reservation.

## Ethics Statement

The studies involving human participants were reviewed and approved by the Comitato Etico Area Vasta Centro (CEAVC). The patients/participants provided their written informed consent to participate in this study.

## Author Contributions

EB, CM, AB, and SC contributed to the conception and design of the study. EB, LM, LL, and SA enrolled, acquired, and preprocessed data for the work. CM and EP executed the ML and the DL analyses, respectively. EB, LM, CM, and EP wrote the first draft of the manuscript. MB, AB, SC, LG, MAP, and VM revised it critically for important intellectual content. All authors contributed to manuscript revision, read, and approved the submitted version.

## Funding

This work received fundings for open access publication fees from NAVIGATOR project, Bando Ricerca Salute 2018, Regione Toscana (http://navigator.med.unipi.it/).

## Conflict of Interest

The authors declare that the research was conducted in the absence of any commercial or financial relationships that could be construed as a potential conflict of interest.

The handling editor declared a past co-authorship with one of the authors VM.

## Publisher’s Note

All claims expressed in this article are solely those of the authors and do not necessarily represent those of their affiliated organizations, or those of the publisher, the editors and the reviewers. Any product that may be evaluated in this article, or claim that may be made by its manufacturer, is not guaranteed or endorsed by the publisher.
