# Supplementary material for: Machine and Deep Learning Prediction Of Prostate Cancer Aggressiveness Using Multiparametric MRI
Source: Front Oncol. 2022 Jan 13;11:802964. doi: 10.3389/fonc.2021.802964 (PMC8792745; doi:10.3389/fonc.2021.802964)
Supplement: Supplementary file 1 [file DataSheet_1.pdf]

Supplementary Material

1 MATERIALS AND METHODS

1.1 MRI examinations

All mpMRI examinations were performed using a 1.5 T MR scanner equipped with an anterior pelvic phased-array 18-channel coil and a posterior spine phased-array 16-channel coil (Magnetom Aera, Siemens Medical Systems, Erlangen, Germany). The PI-RADS 2.0 acquisition protocol included high-resolution T2-weighted (T2w) sequences in the axial (TR/TE = 4150/123 ms, voxel size = 0.6 × 0.6 × 3.0 mm^3), sagittal (TR/TE = 3850/101 ms, voxel size = 0.7 × 0.7 × 3.0 mm^3) and coronal (TR/TE = 3210/123 ms, voxel size = 0.7 × 0.7 × 3.0 mm^3) planes; a T1-weighted sequence (TR/TE = 450/10 ms, voxel size = 0.6 x 0.6 x 3.0 mm) in the axial plane; a multi-b Diffusion Weighted Imaging (DWI) (b values = [0, 500, 1000, 1500, 2000] s/mm^2), voxel size = 0.8 × 0.8 × 3 mm, three directions) echo-planar imaging (EPI) sequence from which corresponding ADC maps were automatically calculated using software on board of the MRI console, and a Dynamic Contrast Enhancement (DCE) assessment with time intensity curves evaluation.
The PI-RADS 2.1 acquisition protocol included high-resolution T2w sequences in the axial (TR/TE = 4790/123 ms, voxel size = 0.3 x 0.3 x 3.0 mm^3), sagittal (TR/TE = 4470/101 ms, voxel size = 0.3 x 0.3 x 3.0 mm^3) and coronal (TR/TE = 3520/123 ms, voxel size = 0.3 x 0.3 x 3.0 mm^3) planes, automatically interpolated from a voxel size of 0.74 x 0.63 x 3.00 mm^3 by the MRI console; a T1-weighted sequence (TR/TE = 450/10 ms, voxel size = 0.6 x 0.6 x 3.0 mm^3) in the axial plane; a multi-b DWI (b values = [50, 100, 800, 1000] s/mm^2, voxel size = 1.0 x 1.0 x 3.0 mm^3, three directions) EPI sequence, automatically interpolated from a voxel size of 2.60 x 2.08 x 3.00 mm^3 by the MRI console, whose corresponding ADC maps were automatically calculated using software on board of the MRI console; a high-b DWI (b values: [1400, 1800] s/mm^2, voxel size = 2.2 x 2.2 x 3.0 mm^3, three directions) EPI sequence, and a Dynamic Contrast Enhancement (DCE) assessment with time intensity curves evaluation.

1.2 Experimental tests

We manually segmented tumor areas independently on T2w images and ADC maps by 3D Slicer software v. 4.10.2 (Fedorov et al., 2012) on a Dual-Core Intel Core i5 MacBook Air with 16 GB RAM. Briefly, we have outlined the segmentation on each DICOM slice containing the tumoral area. Then, we saved the entire 3D lesion segmentation and the original image, i.e., T2w image and ADC map, in the .NRRD format.
For the ML algorithms, we extracted single slices from each lesion segmentation by using custom code in Python language (v. 3.9.2) and the following libraries: pynrrd (v. 0.4.2) (`https://pypi.org/project/pynrrd/`), numpy (v. 1.21.0.dev0+1518.ge3583316c) (Harris et al., 2020), and matplotlib (v. 3.4.2) (Hunter, 2007). The workstation used is a Dual-Core Intel Core i7 MacBook with 16 GB RAM. We extracted radiomics features through pyradiomics (v. 3.0.1) (Griethuysen et al., 2017), on a Linux virtual machine (Ubuntu v. 18.04.3) with 4 CPU cores and 8 GB RAM, hosted on a Dell PowerEdge R540 workstation equipped with 32 logical Intel(R) Xeon(R) Silver 4108 CPU cores. In particular, we forced a customized 2D extraction (details in Table S1). The ML frameworks' training, validation, and test were carried out using a custom code in Python language using the following modules: imbalanced-learn (v. 0.8.0) (Lemaitre et al., 2017), matplotlib (v. 3.4.2) (Hunter, 2007), numpy (v. 1.21.0.dev0+1518.ge3583316c) (Harris et al., 2020), pandas (v. 1.2.4) (Reback et al., 2021), scikit-learn (v. 1.0.dev0) (Pedregosa et al., 2011), xgboost (v. 1.4.2) (Chen et al., 2016). In particular, we used

BaggingClassifier, *RandomForestClassifier*, and *ExtraTreeClassifier* estimators for the ensemble averaging methods, while *AdaBoostClassifier*, *GradientBoostingClassifier*, and *XGBClassifier* as boosting algorithms. A different combination of hyperparameters for each estimator was tuned in the validation set. The total computation time for the training, validation, and test was about three days on a single core of a Dual-Core Intel Core i7 MacBook with 16 GB RAM.
Regarding the DL experimentation, we performed DICOM slice selection using 3D Slicer software (Fedorov et al., 2012), and the PNG input images were retrieved from DICOM files using pydicom (v. 2.1.2) (Mason, 2011) and pillow (v. 8.3.2) (Clark, 2015) libraries. Images containing the tumor lesion alone were obtained exploiting pynrrd module (v. 0.4.2), and the rotated, translated, and flipped version of each image was generated using torchvision library (v. 0.9.1) (Paszke et al., 2019). The workstation used is an Intel Core i7 ASUS Laptop with 8 GB RAM.
As for ML frameworks, DL ones were developed in Python language using a custom code based on the following libraries: matplotlib (v. 3.4.2) (Hunter, 2007), numpy (v. 1.22.0.dev0+4.gb283e1632) (Harris et al., 2020), pandas (v. 1.2.4) (Reback et al., 2021), pytorch (v. 1.8.1) (Paszke et al., 2019), scikit-learn (v. 1.0.dev0) (Pedregosa et al., 2011). Several CNN architectures were developed, and for each one, hyperparameters tuning was carried out. Using an Intel Core i7 ASUS Desktop Computer with 32 GB RAM and exploiting the integrated GPU NVIDIA GeForce GTX 1650, each tuning process required between a half-day and two days, according to the architecture complexity.

1.3 Radiomics features extraction

For each slice, a total of 95 features were obtained (details in Table S2): 9 2D-shape features, 18 first-order features, and 68 second-order features (i.e., textural features) from grey level co-occurrence matrix (GLCM, 22 features), grey level run length matrix (GLRLM, 16 features), grey level size zone matrix (GLSZM, 16 features), and grey level dependence matrix (GLDM, 14 features). Second-order features estimation was performed according to the Chebyshev norm with a distance of 1 pixel. We have computed all radiomic features in compliance with the Image Biomarker Standardisation Initiative (IBSI). It is worth noting that the first-order feature of Kurtosis was IBSI-compliant except for an offset value (i.e., 3).

1.4 DL analysis: CNN architectures

A grid search approach has been adopted to test 30 different architectures. The depth of the architecture has been evaluated by varying from three to seven convolutional blocks. Each network starts with its first block, made of two 3x3 kernel convolutional layers, an activation, and a batch normalization layer. The subsequent blocks consist of a sequence of a 1x1 and a 3x3 kernel convolutional layers, an activation, and a batch normalization layer. We have placed a max-pooling layer every two convolutional blocks to introduce spatial invariance and reduce computational time. Eventually, we tested the final layers of the architecture using one, two, or three fully connected layers. The last block is always composed of a fully connected layer and a dropout layer. The other block (one or two blocks before the last one) consists of a fully connected layer, an activation layer, and a dropout layer. For each architecture, we evaluated both ReLU and LeakyReLU activation layers. A schematic description of the grid search is provided in Table S4, while the description of the different blocks (type and number) can be found in Table S5. We performed a hyperparameter optimization for each architecture, searching for the learning rate values, the weights decay, the dropout probability, the batch size, and whether to choose AMSGrad (Reddi et al., 2019), a variant of the Adam optimizer (Kingma et al., 2014). To limit computational time, we randomly chose 50 configurations to test, each one consisting of a specific combination of the values of the five hyperparameters mentioned above. We have reported the values assumed by the hyperparameters during the random search in Table

S6. Moreover, we added two Attention Gates (AGs) to the three optimal architectures trained on C-DS T2w/ADC/T2w+ADC images (Schlemper et al., 2019). According to (Schlemper et al., 2019), AG modules are more efficient when placed on layers handling higher-level and more specific features; hence, we tested different placements for the AG modules, considering only the middle and the final layers of the architecture. Finally, an additional hyperparameter optimization was applied to each of them.

1.5 Training, validation, and test of ML/DL frameworks

The ML/DL frameworks have been trained, validated and tested following a patient-based nested validation scheme: data of 87% of patients formed the development set and data of 13% of patients were included in the test set. In the development set only, we adopted a 5-fold cross-validation (CV) (4 folds were used as training set while the other one as the validation set), because it offers a favorable bias-variance trade-off (Hastie et al., 2009; Lemm et al., 2011) and is also adequate for frameworks selection (Breiman et al., 1992). In creating the CV folds, images splitting was done on a patient basis, i.e., a unique fold contained a patient and all his images. Following a stratified-group procedure, the relative proportion of LG and HG data was preserved within each fold. For DL architectures, the training set was further randomly divided (90% to train the network and 10% to validate the early stopping criterion). The criterion was as follows: when the loss during the validation phase exceeded the loss during the training phase for more than three consecutive epochs, the training is stopped. We trained the network for a maximum number of epochs equal to 100 to limit the computational complexity of the training process. The CV procedure has been repeated ten times using different random splits to deal with the variability in framework and hyperparameters selection derived from a specific data split (Krstajic et al., 2014). We have computed the average and standard deviation of the Area Under the Receiver Operating curve (AUROC) across all repetitions to get the final scores. The best frameworks were chosen based on the average AUROC scores in the validation set and retrained on the whole development set. For the DL retraining, to prevent overfitting, we retrained the best architectures (one for each acquisition modality), keeping a 10% of the development set as validation, maintaining patient separation and stratification across classes. We applied early stopping when validation loss did not decrease or change for five consecutive epochs. Since the retraining is less time-consuming than the grid search optimization, the number of epochs was increased to 1000.

REFERENCES

Breiman L, Spector P. Submodel Selection and Evaluation in Regression. The X-Random Case. International Statistical Review / Revue Internationale de Statistique. 1992;60(3):291. doi:10.2307/1403680

Chen T, Guestrin C. XGBoost: A Scalable Tree Boosting System. Proceedings of the 22nd ACM SIGKDD International Conference on Knowledge Discovery and Data Mining. 2016:785–794. doi:10.1145/2939672.2939785

Clark A. Pillow (PIL Fork) Documentation. 2015.

Fedorov A, Beichel R, Kalpathy-Cramer J, et al. 3D Slicer as an image computing platform for the Quantitative Imaging Network. Magn Reson Imaging. 2012;30(9):1323-1341. doi:10.1016/j.mri.2012.05.001

Harris CR, Millman KJ, van der Walt SJ, et al. Array programming with NumPy. Nature. 2020;585(7825):357-362. doi:10.1038/s41586-020-2649-2

Hastie T, Tibshirani R, Friedman J. The Elements Of Statistical Learning. Springer; 2009.

Hunter J. Matplotlib: A 2D Graphics Environment. Comput Sci Eng. 2007;9(3):90-95. doi:10.1109/mcse.2007.55.

Kingma D, Ba J. Adam: A Method for Stochastic Optimization. ICLR (Poster). 2015.

Krstajic D, Buturovic LJ, Leahy DE, Thomas S. Cross-validation pitfalls when selecting and assessing regression and classification models. J Cheminform. 2014;6(1):10. Published 2014 Mar 29. doi:10.1186/1758-2946-6-10

Lemaitre G, Nogueira F, Aridas C. Imbalanced-learn: A Python Toolbox to Tackle the Curse of Imbalanced Datasets in Machine Learning. Journal of Machine Learning Research. 2017;18(1):559–563.

Lemm S, Blankertz B, Dickhaus T, Müller KR. Introduction to machine learning for brain imaging. Neuroimage. 2011;56(2):387-399. doi:10.1016/j.neuroimage.2010.11.004

Mason D. SU-E-T-33: Pydicom: An Open Source DICOM Library. Med Phys. 2011;38(6Part10):3493-3493. doi:10.1118/1.3611983

Paszke A, Gross S, Massa F, Lerer A, Bradbury J. PyTorch: An Imperative Style, High-Performance Deep Learning Library. Adv Neural Inf Process Syst. 2019;32:8024–8035.

Pedregosa F, Varoquaux G, Gramfort A, Michel V. Scikit-learn: Machinelearning in Python. Journal of Machine Learning Research. 2011;12:2825–2830.

Reback J, McKinney W, Bossche J et al. pandas-dev/pandas: Pandas 1.3.4. Zenodo. https://doi.org/10.5281/zenodo.35[illegible] Published 2021.

Reddi S, Kale S, Kumar S. On the Convergence of Adam and Beyond. In: ICLR 2018 Conference. 2018; arXiv:1904.09237(2019).

van Griethuysen JJM, Fedorov A, Parmar C, et al. Computational Radiomics System to Decode the Radiographic Phenotype. Cancer Res. 2017;77(21):e104-e107. doi:10.1158/0008-5472.CAN-17-0339

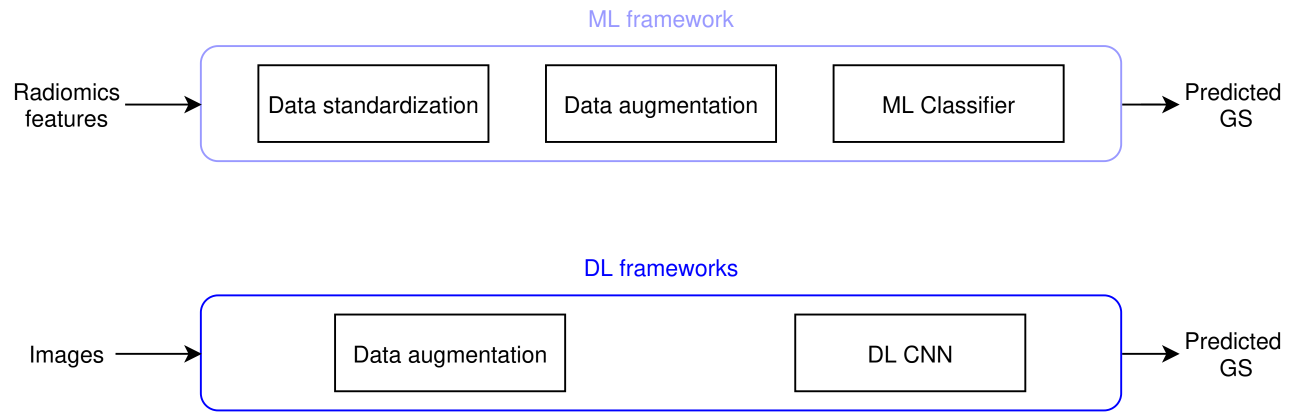

Figure S1. ML/DL frameworks.

the framework trained with radiomic features extracted from T2w images

Table S1. YAML parameter file for the radiomics features extraction through pyradiomics.

Category	Parameter	Value
imageType	Original	{}
featureClass	shape2D	[]
	firstorder	[]
	glcm	- 'Autocorrelation'
		- 'JointAverage'
		- 'ClusterProminence'
		- 'ClusterShade'
		- 'ClusterTendency'
		- 'Contrast'
		- 'Correlation'
		- 'DifferenceAverage'
		- 'DifferenceEntropy'
		- 'DifferenceVariance'
		- 'JointEnergy'
		- 'JointEntropy'
		- 'Imc1'
		- 'Imc2'
		- 'Idm'
		- 'Idmn'
		- 'Id'
		- 'Idn'
		- 'InverseVariance'
		- 'MaximumProbability'
		- 'SumEntropy'
		- 'SumSquares'
	glrlm	[]
	glszm	[]
	gldm	[]
Setting	normalize	False
	normalizeScale	1
	removeOutliers	None
	force2D	True
	force2Ddimension	0
	binWidth	25
	label	1
	interpolator	'sitkBSpline'
	resampledPixelSpacing	None
	weightingNorm	None
	minimumROIDimensions	2
	minimumROISize	None
	preCrop	False
	padDistance	5
	distances	[1]
	resegmentRange	None
	additionalInfo	True
	correctMask	True

Table S2. Radiomics features. Glcm: Gray Level Co-occurrence Matrix; glrlm: Gray Level Run Length Matrix; glszm: Gray Level Size Zone Matrix; gldm: Gray Level Dependence Matrix.

Category	Feature
shape 2D	Elongation, MajorAxisLength, MaximumDiameter, MeshSurface, MinorAxisLength, Perimeter, PerimeterSurfaceRatio, PixelSurface, Sphericity
first order	10Percentile, 90Percentile, Energy, Entropy, InterquartileRange, Kurtosis, Maximum, MeanAbsoluteDeviation, Mean, Median, Minimum, Range, RobustMeanAbsoluteDeviation, RootMeanSquared, Skewness, TotalEnergy, Uniformity, Variance
glcm	Autocorrelation, JointAverage, ClusterProminence, ClusterShade, ClusterTendency, Contrast, Correlation, DifferenceAverage, DifferenceEntropy, DifferenceVariance, JointEnergy, JointEntropy, Imc1, Imc2, Idm, Idmn, Id, Idn, InverseVariance, MaximumProbability, SumEntropy, SumSquares
glrlm	GrayLevelNonUniformity, GrayLevelNonUniformityNormalized, GrayLevelVariance, HighGrayLevelRunEmphasis, LongRunEmphasis, LongRunHighGrayLevelEmphasis, LongRunLowGrayLevelEmphasis, LowGrayLevelRunEmphasis, RunEntropy, RunLengthNonUniformity, RunLengthNonUniformityNormalized, RunPercentage, RunVariance, ShortRunEmphasis, ShortRunHighGrayLevelEmphasis, ShortRunLowGrayLevelEmphasis
glszm	GrayLevelNonUniformity, GrayLevelNonUniformityNormalized, GrayLevelVariance, HighGrayLevelZoneEmphasis, LargeAreaEmphasis, LargeAreaHighGrayLevelEmphasis, LargeAreaLowGrayLevelEmphasis, LowGrayLevelZoneEmphasis, SizeZoneNonUniformity, SizeZoneNonUniformityNormalized, SmallAreaEmphasis, SmallAreaHighGrayLevelEmphasis, SmallAreaLowGrayLevelEmphasis, ZoneEntropy, ZonePercentage, ZoneVariance
gldm	DependenceEntropy, DependenceNonUniformity, DependenceNonUniformityNormalized, DependenceVariance, GrayLevelNonUniformity, GrayLevelVariance, HighGrayLevelEmphasis, LargeDependenceEmphasis, LargeDependenceHighGrayLevelEmphasis, LargeDependenceLowGrayLevelEmphasis, LowGrayLevelEmphasis, SmallDependenceEmphasis, SmallDependenceHighGrayLevelEmphasis, SmallDependenceLowGrayLevelEmphasis

Table S3. ML frameworks' hyperparameters.

Estimator	Hyperparameters
AdaBoostClassifier	n_estimators=[10, 50, 100], learning_rate=[0.1, 1.0, 10], algorithm='SAMME.R', random_state=0
BaggingClassifier	n_estimators=[10, 50, 100, 1000], max_samples=[0.5, 0.8, 1.0], max_features=[0.5, 0.8, 1.0], bootstrap=False, bootstrap_features=False, oob_score=False, warm_start=False, n_jobs=None, random_state=None, verbose=0
ExtraTreeClassifier	n_estimators=[10, 50, 100], criterion='gini', max_depth=[None, 2, 3, 4, 5], min_samples_split=2, min_samples_leaf=1, min_weight_fraction_leaf=0.0, max_features='auto', max_leaf_nodes=None, min_impurity_decrease=0.0, min_impurity_split=None, bootstrap=False, oob_score=False, n_jobs=None, random_state=None, verbose=0, warm_start=False, class_weight=None, ccp_alpha=0.0, max_samples=None
GradientBoostingClassifier	loss='deviance', learning_rate=[0.1, 1.0, 10], n_estimators=[10, 50, 100], subsample=1.0, criterion='friedman_mse', min_samples_split=2, min_samples_leaf=1, min_weight_fraction_leaf=0.0, max_depth=3, min_impurity_decrease=0.0, min_impurity_split=None, init=None, random_state=0, max_features=None, verbose=0, max_leaf_nodes=None, warm_start=False, validation_fraction=0.1, n_iter_no_change=None, tol=0.0001, ccp_alpha=0.0
RandomForestClassifier	n_estimators=[10, 100, 1000], criterion='gini', max_depth=[None, 2, 3, 4, 5], min_samples_split=2, min_samples_leaf=1, min_weight_fraction_leaf=0.0, max_features='auto', max_leaf_nodes=None, min_impurity_decrease=0.0, min_impurity_split=None, bootstrap=False, oob_score=False, n_jobs=None, random_state=None, verbose=0, warm_start=False, class_weight=None, ccp_alpha=0.0, max_samples=None
XGBClassifier	gamma=[0, 0.1, 1, 10, 100], learning_rate=[0.01, 0.1, 0.3], max_depth=[2, 3], n_estimators=[10, 50, 100], importance_type='gain', objective=binary:logistic, verbosity=0, subsample=0.5

Table S4. Composition of blocks used in Grid Search to select the most promising DL network architecture.

Layer name	Composition
Convolutional Block 1	3x3 conv layer<br>3x3 conv layer<br>activation layer<br>batch normalization layer
Convolutional Block 2	1x1 conv layer<br>activation layer<br>3x3 conv layer<br>activation layer<br>batch normalization layer
Fully Connected Block 1	fully connected layer<br>activation layer<br>dropout layer
Fully Connected Block 2	fully connected layer<br>dropout layer

Table S5. Number and type of the experimented blocks used in Grid Search to select the most promising DL network architecture.

Layer name	Number and type
Convolutional Block 1	1 (always present at the beginning)
Convolutional Block 2	2 to 7
Fully Connected Block 1	1 to 2
Fully Connected Block 2	1 (always present at the end)
Activation	ReLU and Leaky ReLU

Table S6. DL networks hyperparameters values in the Random Search to select the most promising DL network architecture.

Hyperparameter	Values
Learning rate	0.01<br>0.001<br>0.0001
Weight decay	0.1<br>0.01<br>0.001
Dropout probability	0.5<br>0.6<br>0.7<br>0.8
Amsgrad	True<br>False
Batch size	4<br>8

Table S7. AUROC values of ML and DL analyses for T2w/ADC/T2w+ADC. The AUROC values in the validation set are reported as mean (standard deviation), while the AUROC values in the test set 2.1 are reported as median [5th percentile, 95th percentile]. AG: attention gate; C-DS: cropped dataset; DL: deep learning; L-DS: lesion dataset; ML: machine learning.

Framework	Set	T2w	ADC	T2w+ADC
ML	Validation	0.728 (0.02)	0.776 (0.02)	0.748 (0.01)
	Test 2.1	0.357 [0.200, 0.495]	0.385 [0.231, 0.529]	0.558 [0.375, 0.712]
AG-free DL on L-DS	Validation	0.709 (0.03)	0.645 (0.02)	0.658 (0.02)
	Test 2.1	0.670 [0.492, 0.815]	0.517 [0.410, 0.634]	0.650 [0.434, 0.855]
AG-free DL on C-DS	Validation	0.716 (0.03)	0.637 (0.03)	0.694 (0.04)
	Test 2.1	0.299 [0.216, 0.467]	0.380 [0.324, 0.479]	0.406 [0.239, 0.607]
AG DL on C-DS	Validation	0.634 (0.03)	0.607 (0.06)	0.584 (0.09)
	Test 2.1	0.234 [0.155, 0.382]	0.463 [0.311, 0.675]	0.480 [0.310, 0.633]

Table S8. Best performing ML frameworks selected on the average AUROC value in the PI-RADS 2.0 validation set.

	T2w	ADC	T2w+ADC
Data standardizer	Standard scaler (with_mean=True, with_std=True)	Standard scaler (with_mean=True, with_std=True)	Standard scaler (with_mean=True, with_std=True)
Data augmentizer	SVMSMOTE (random_state=9)	SVMSMOTE (random_state=9)	BorderlineSMOTE (random_state=9)
Data classifier	AdaBoost classifier (hyperparameters: base_estimator= None, n_estimators=50, learning_rate=0.1, algorithm= 'SAMME.R', random_state=0)	XGBoost classifier (hyperparameters: gamma=10, learning_rate=0.01, max_depth=2, n_estimators=100, importance_type= 'gain', objective = 'binary:logistic', verbosity=0, subsample=0.5)	Extra Trees classifier (hyperparameters: n_estimators=100, criterion='gini', max_depth=2, min_samples_split= 2, min_samples_leaf= 1, min_weight_fraction_leaf= 0.0, max_features= 'auto', max_leaf_nodes= None, min_impurity_decrease= 0.0, min_impurity_split= None, bootstrap= False, oob_score= False, n_jobs= None, random_state= None, verbose= 0, warm_start= False, class_weight= None, ccp_alpha= 0.0, max_samples= None)

Table S9. Best performing DL frameworks selected on the average AUROC value in the PI-RADS 2.0 validation set. BS = batch size; DP = dropout probability; LR = learning rate; WD = weight decay.

		T2w	ADC	Multimodal
AG-free DL on L-DS	Architecture	5 convolutional blocks, 2 max pooling 3 fully connected blocks, ReLU activation function	3 convolutional blocks, 1 max pooling 3 fully connected blocks, ReLU activation function	Ensemble of the two optimal architectures fed with T2w images and ADC maps, respectively
	Hyperparameters	LR = 0.0001, WD = 0.01, Amsgrad = False, DP = 0.5, BS = 4	LR = 0.0001, WD = 0.01, Amsgrad = False, DP = 0.8, BS = 8	LR = 0.0001, WD = 0.01, Amsgrad = False, DP (t2 branch) = 0.5, DP (adc branch) = 0.8, BS = 8
AG-free DL on C-DS	Architecture	4 convolutional blocks, 2 max pooling 3 fully connected blocks, ReLU activation function	3 convolutional blocks, 1 max pooling, 3 fully connected blocks and ReLU activation function	Ensemble of the two optimal architectures fed with T2w images and ADC maps, respectively
	Hyperparameters	LR = 0.0001, WD = 0.01, Amsgrad = True, DP = 0.8, BS = 4	LR = 0.0001, WD = 0.01, Amsgrad = False, DP = 0.8, BS = 8	LR = 0.0001, WD = 0.01, Amsgrad = False, DP (both branches) = 0.8, BS = 4
AG DL on C-DS	Architecture	4 convolutional blocks, 2 max pooling 3 fully connected blocks, ReLU activation function. Two AGs placed on the second and third convolutional layers.	3 convolutional blocks, 1 max pooling, 3 fully connected blocks and ReLU activation function. Two AGs placed on the last two convolutional layers	Ensemble of the two optimal architectures fed with T2w images and ADC maps, respectively
	Hyperparameters	LR = 0.0001, WD = 0.01, Amsgrad = True, DP = 0.8, BS = 4	LR = 0.0001, WD = 0.01, Amsgrad = False, DP = 0.8, BS = 8	LR = 0.0001, WD = 0.01, Amsgrad = True, DP (both branches) = 0.8, BS = 8

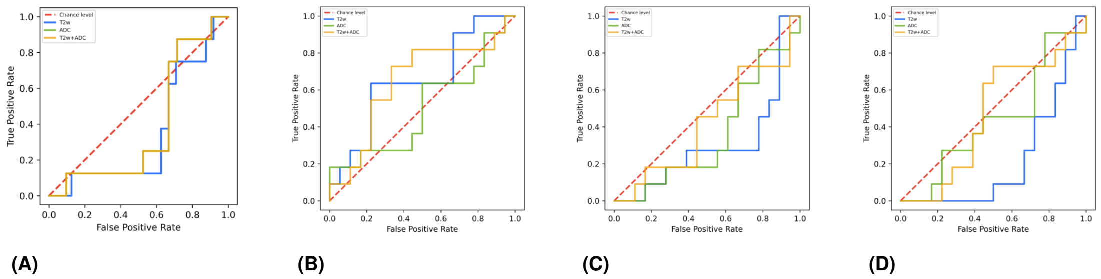

Figure S2. **(A)** ROC curves of ML frameworks on the test set 2.1. **(B)** ROC curves of DL AG-free CNN trained on L-DS test set 2.1. **(C)** ROC curves of DL AG-free CNN trained on C-DS test set 2.1. **(D)** ROC curves of DL AG CNN trained on C-DS test set 2.1.

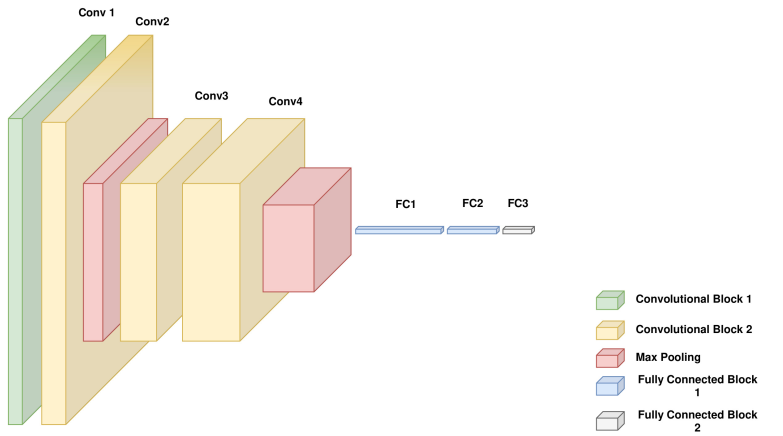

Figure S3. Optimal AG-free CNN architecture, trained on C-DS T2w images.

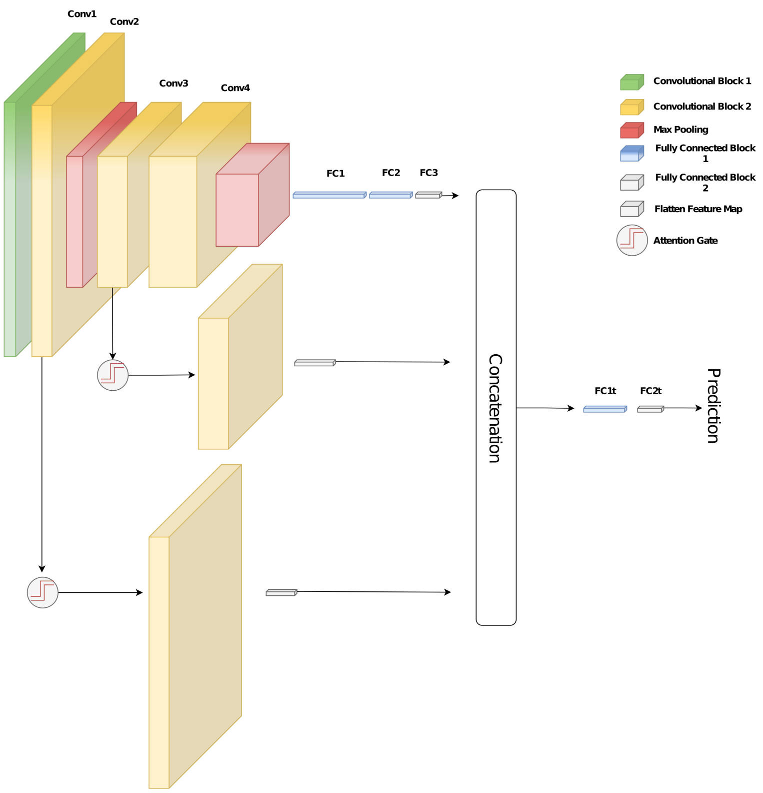

Figure S4. Optimal AG CNN architecture, trained on C-DS T2w images.

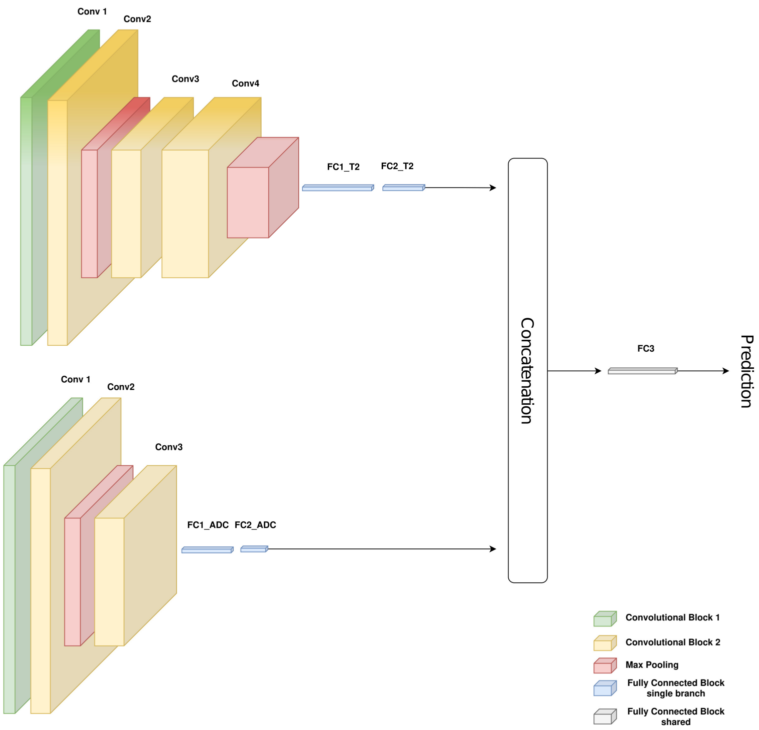

Figure S5. Optimal AG-free CNN architecture, trained on C-DS T2w images and ADC maps.

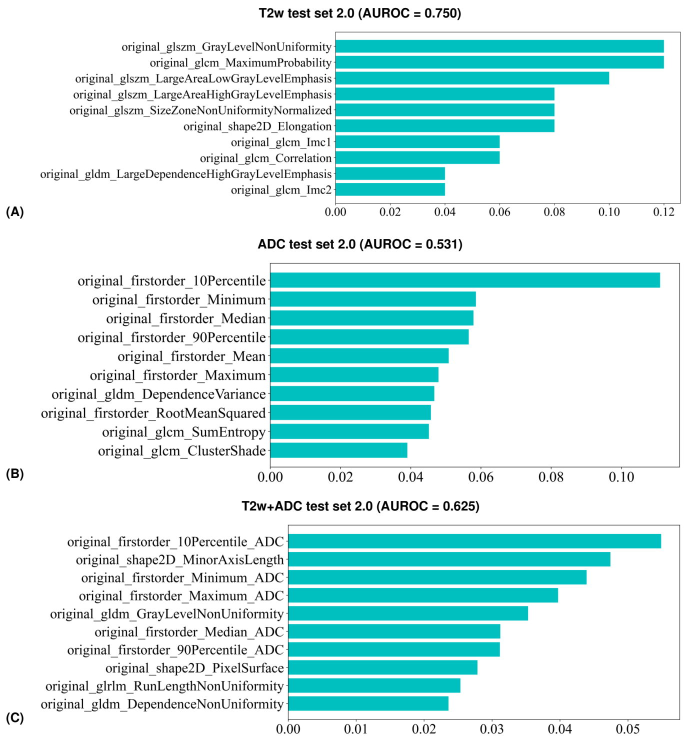

Figure S6. Bar plots of feature importance of ML frameworks trained on development set 2.0. The ML frameworks were trained with radiomic features extracted from T2w images **(A)**, ADC maps **(B)**, and T2w images + ADC maps **(C)** respectively.

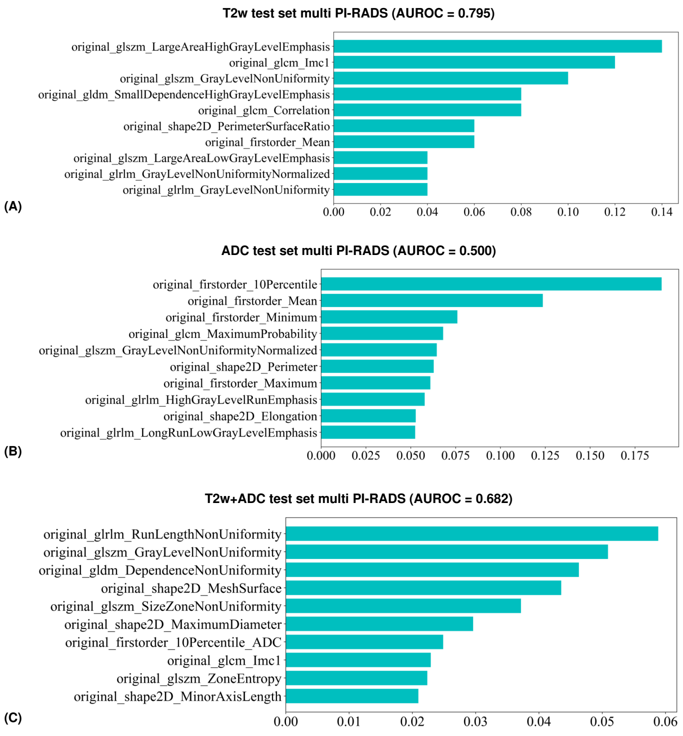

Figure S7. Bar plots of feature importance of ML frameworks trained on multi PI-RADS development set. The ML frameworks were trained with radiomic features extracted from T2w images **(A)**, ADC maps **(B)**, and T2w images + ADC maps **(C)** respectively.
